# Case-Control Study of Household and Environmental Transmission of Typhoid Fever in India

**DOI:** 10.1093/infdis/jiab378

**Published:** 2021-11-23

**Authors:** Sidhartha Giri, Venkata Raghava Mohan, Manikandan Srinivasan, Nirmal Kumar, Vinoth Kumar, Pavithra Dhanapal, Jayalakshmi Venkatesan, Annai Gunasekaran, Dilip Abraham, Jacob John, Gagandeep Kang

**Affiliations:** 1 Division of Gastrointestinal Sciences, Christian Medical College, Vellore, India; 2 Department of Community Health, Christian Medical College, Vellore, India

**Keywords:** environment, India, risk factors, *Salmonella*, typhoid

## Abstract

**Background:**

Typhoid fever causes substantial morbidity and mortality in low- and middle-income countries. We conducted a case-control study in Vellore, southern India, to understand risk factors for transmission of typhoid.

**Methods:**

From April 2018 to October 2019, households of blood culture-confirmed typhoid cases that occurred within a fever surveillance cohort aged 6 months–15 years, and controls matched for age, sex, geographic location, and socioeconomic status, were recruited. Information on risk factors was obtained using standard questionnaires. Household and environmental samples were collected for detection of *Salmonella* Typhi using real-time polymerase chain reaction. Multivariable analysis was used to evaluate associations between risk factors and typhoid.

**Results:**

One hundred pairs of cases and controls were recruited. On multivariable regression analysis, mothers eating food from street vendors during the previous week (odds ratio [OR] = 2.04; 95% confidence interval [CI], 1.03–4.12; *P = *.04) was independently associated with typhoid, whereas treatment of household drinking water (OR = 0.45; 95% CI, 0.25–0.80; *P = *.007) was protective. There was no significant difference in *S* Typhi detection between the environmental samples from case and control households.

**Conclusions:**

Street-vended food is a risk factor for typhoid in densely populated urban communities of Vellore. Improved sanitation facilities and awareness about point-of-use water treatment are likely to contribute to typhoid control.

Typhoid fever, caused by the bacterium *Salmonella* serovar Typhi, is a cause of substantial morbidity and mortality in low-income and middle-income countries, causing approximately 11.9 million cases and 1 29 000 deaths annually [1]. *Salmonella* Typhi is a strictly human pathogen and is transmitted through the fecal-oral route.

A recent systematic review on association of typhoid fever with water, sanitation, hygiene, and food exposures in case-control studies, which included endemic and outbreak settings, found that factors such as surface water contact, untreated water, unsafe waste management, lack of hygiene, risky food practices, food and drink outside the home, and specific foods such as dairy, ice cream, fruit and juice, were significant risk factors for typhoid fever [2]. However, there is heterogeneity in the published data regarding the ecological niche of *S* Typhi in the environment and the risk factors for the transmission of typhoid. *Salmonella* Typhi was detected in the water for irrigation of salad vegetables in Santiago, Chile and from a substantial proportion of municipal drinking water samples in Kathmandu, Nepal [3, 4]. Other risk factors for transmission of typhoid can potentially include household drinking water and food [2].

There is a need for contemporaneous studies to better understand current environmental risk factors for transmission of *S* Typhi. This has assumed critical importance after the emergence of cephalosporin-resistant *S* Typhi in Pakistan, limiting available antibiotics for treatment of typhoid fever [5]. In addition to the introduction of the typhoid conjugate vaccine for disease control, identifying environmental risk factors for typhoid transmission and focusing on interventions to curtail these transmission pathways will play a crucial role in the control of typhoid fever.

In this case-control study, we evaluated the risk factors for environmental transmission of typhoid fever in Vellore, in the southern state of Tamil Nadu, during 2018 to 2019.

## METHODS

### Study Setting

**Figure 1.**
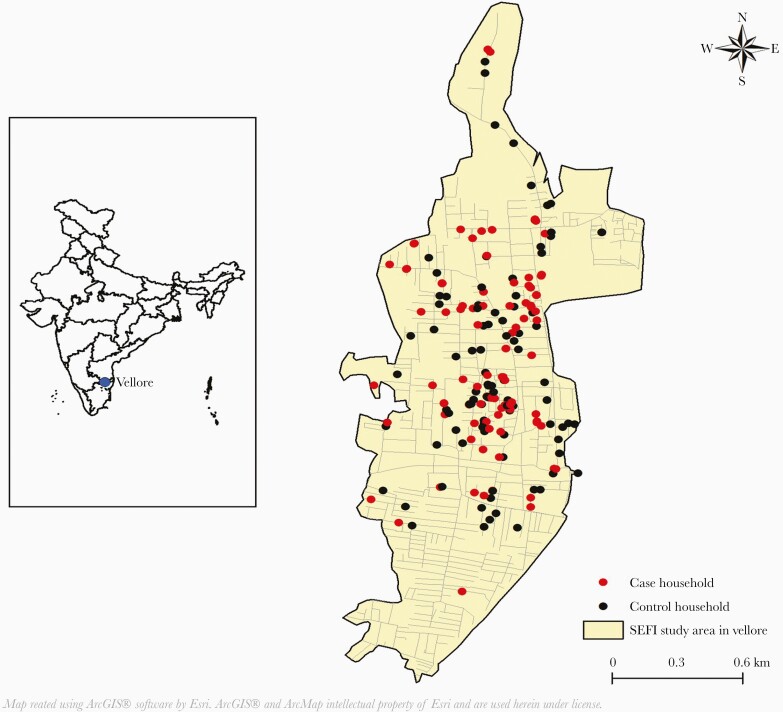
Spatial distribution of typhoid cases and control households in Vellore. SEFI, Surveillance for Enteric Fever in India.

The case-control study was conducted between April 1, 2018 and October 5, 2019. Vellore city (12.92°N 79.13°E) is the administrative headquarters of Vellore district, located on the Palar riverbank in northeastern Tamil Nadu, India (Figure 1). Vellore has 4 zones (totally 60 wards) that cover 87.915 km^2^ and a population of approximately 500 000 based on the 2011 Government of India census. The city has a semiarid climate with high temperatures throughout the year and relatively low rainfall. There are 3 seasons: summer (March–July, temperatures >40°C), rains (August–November, with both southwest and northeast monsoons), and winter (December–February, low of 15°C). The average total rainfall per year is 1053 mm; approximately 60% occurs during the rainy season.

### Typhoid Fever Surveillance

Between October 2017 and December 2019, the Surveillance for Enteric Fever in India (SEFI) study was carried out at 4 sites in India: Delhi (North), Kolkata (East), Pune (West), and Vellore (South), to estimate the incidence rate of blood culture-confirmed typhoid fever in children aged between 6 months and 15 years. The SEFI study protocol has been described in detail previously [6, 7]. At each site, a cohort of approximately 6000 children was followed up with weekly surveillance, either by a home visit or over the phone to obtain information about febrile episodes. In Vellore, the cohort was established in the contiguous semiurban settlements of Chinallapuram, Kaspa, Ramanaickanpalayam, and Vasanthapuram, which are part of the Vellore Demographic Surveillance System [7]. *“*Suspected typhoid fever” included any febrile episode with fever for ≥3 days, with the child continuing to be febrile over the last 12 hours. Blood culture was performed for all suspected cases. If the blood culture was positive for typhoid fever, the child was assessed by a study physician and either treated with oral azithromycin or referred if the episode warranted hospitalization.

### Case-Control Study

This case-control study was nested within the SEFI cohort. A case was defined as a child aged 6 months to 15 years with blood culture-confirmed typhoid fever. For every case, a control was selected by matching for age, sex, geographic location, type of housing, and socioeconomic status, but with no history of fever in the family during the past 1 month. Based on the Vellore census data, the control household was chosen randomly from the list of eligible control households near the case household, after obtaining written informed consent from the parents. A household questionnaire collected data on potential risk factors from mothers of both cases and controls. The GPS location of the households of the cases and controls were captured using GPSMAP 62 (Garmin Ltd., Olathe, KS). All of the GPS readings of the households of cases and controls were recorded and visualized using the ArcGIS software (ESRI, Redlands, CA).

### Collection of Environmental Samples and Laboratory Testing

From the households of cases, environmental samples were collected within 72–96 hours of case confirmation. For every case, a matched control was recruited within 3–4 days, and the samples were collected within the next 24–48 hours. From each household, mother and child (case or control) hand rinse, drinking water, and floor swab samples were collected according to the SaniPath protocol [8]. For the sewage outlet draining from each house, a Moore swab sample was placed for 48–72 hours as per published methods [3, 9]. The samples were transported at 4°C to the laboratory within 2 hours of collection. All samples were processed following the SaniPath protocol [8]. In brief, the mother and child hand rinse and the drinking water samples were filtered using a 0.45u Filter and the Millipore filtration system (Merck Millipore, Burlington, MA). Each filter was then incubated for 18–24 hours in 10 mL Selenite F broth at 37°C. The Moore swab was incubated overnight for 18–24 hours in Selenite F broth. Deoxyribonucleic acid (DNA) was extracted from 1 mL Selenite F broth using the QIAamp Fast DNA stool mini kit (QIAGEN, Hilden, Germany) and then used for detection of *Salmonella* Typhi in a singleplex real-time polymerase chain reaction (PCR) assay targeting the STY0201 gene of *S* Typhi [10].

In addition to the Moore swab, household sewage samples were collected using the bag-mediated filtration system (BMFS) when large volume samples were accessible from July 2018 onwards [11, 12]. Approximately 5–6 liters of sewage were filtered from each household on site and the filters were brought to the laboratory in controlled temperature carriers [13]. In the laboratory, the pathogens captured on the filter were eluted using beef extract solution as per published methods [11–13]. The eluate was processed by skimmed milk flocculation and pelleted by centrifugation. The pellet was resuspended in 2 mL phosphate-buffered saline. The DNA isolation from the suspension used the QIAamp Fast DNA stool mini kit (QIAGEN), followed by the real-time PCR assay for *S* Typhi [10].

In addition to *S* Typhi, *Salmonella* Paratyphi A and other common nontyphoidal *Salmonella* such as *Salmonella* Typhimurium, *Salmonella* Enteritidis, and a Pan-*Salmonella* target were detected using a multiplex real-time PCR assay [14]. A cycle threshold cutoff value of 35 was used for both the singleplex and multiplex real-time PCR assays. Positive and negative controls were included in each assay run. In addition to real-time PCR assays, 100 mL each of drinking water, mother and child hand rinse samples were used to measure the coliform count.

### Ethics

The study was approved by the Institutional Review Board of Christian Medical College, Vellore (11170 [OBSERVE] dated February 28, 2018). Written informed consent was obtained from the parents before recruitment.

### Statistical Analysis

All data were entered in Excel 2003 (Microsoft). Stata IC/15.1 (StataCorp, College Station, TX) was used for analyses. Odds ratios (ORs) with 95% confidence interval (CI) were measured in a bivariate analysis for the selected variables. A multivariate conditional logistic regression model with all variables with a *P* ≤ .05 in the bivariate analysis was applied to identify the environmental risk factors associated with typhoid fever. A *P* ≤ .05 was considered statistically significant.

## RESULTS

The spatial distribution of the 100 cases and controls is provided in Figure 1. Of the 100 cases/controls, 55 were male. Forthy-seven percent of cases were in children aged >5–10 years (61–120 months), followed by 34% in children aged >10 years (>120 months), and 19% in children aged 0–5 years (6 to ≤60 months). The age (mean ± 1 standard deviation) of the cases was 7.74 ± 3.33 years, compared to 7.73 ± 3.35 years for controls.

The potential risk factors are provided in Table 1. In the bivariate analysis, treatment of household water (OR = 0.43; 95% CI, 0.24–0.75; *P = *.003) and washing produce before consumption (OR = 0.55; 95% CI, 0.31–0.96; *P = *.03) were significantly protective against typhoid fever, whereas the mother eating street food during the previous week was positively associated with typhoid fever (OR = 2.09; 95% CI, 1.07–4.11; *P = *.03). On multivariable regression analysis, the mother eating street food during the previous week (OR = 2.04; 95% CI, 1.03–4.12; *P* = .04) remained independently associated with typhoid fever. Treatment of household water (OR = 0.45; 95% CI, 0.25–0.80; *P* = .007) was associated with lower odds of typhoid fever in the multivariate analysis. Of the 95 households (37 cases, 58 controls) where water was treated, 77.9% (74 of 95) used filtered water, 16.8% (16 of 95) used boiled water, and 5.3% (5 of 95) used reverse osmosis.

**Table 1.** Bivariate and Multivariate Analysis of Risk Factors for Typhoid Fever in Vellore During 2018–2019

Variable				Bivariate Analysis			Multivariate Analysis		
		Case	Control	OR	95% CI	*P* Value	OR	95% CI	*P* Value
Number of people in household	≤5	57	64	0.75	0.42–1.32	.31			
	>5	43	36						
Use of river, ponds, or lakes by mother in the past 1 month	Yes	1	0	3.03	0.12–75.28	.50			
	No	99	100						
Use of river, ponds, or lakes by children in the past 1 month	Yes	2	2	1.00	0.14–7.24	1.00			
	No	98	98						
Use of open drains by mother in the past 1 month	Yes	24	33	0.64	0.34–1.19	.16			
	No	76	67						
Use of open drains by children in the past 1 month	Yes	44	43	1.04	0.60–1.82	.89			
	No	56	57						
Contact with flood water by mother during rains	Yes	37	48	0.64	0.36–1.12	.12			
	No	63	52						
Contact with flood water by children during rains	Yes	36	44	0.72	0.41–1.26	.25			
	No	64	56						
Drinking of municipal water by mother in past 1 week	Yes	97	97	1.00	0.20–5.08	1.00			
	No	3	3						
Drinking of municipal water by children in past 1 week	Yes	97	97	1.00	0.20–5.08	1.00			
	No	3	3						
Treatment of household water (boiling, filtration, reverse osmosis)	Yes	37	58	0.43	0.24–0.75	**.003**	0.45	0.25–0.80	**.007**
	No	63	42						
Number of times bathed per week by mother	≥6	18	16	1.15	0.55–2.41	.71			
	≤5	82	84						
Number of times bathed per week by children	≥6	17	16	1.08	0.51–2.27	.85			
	≤5	83	84						
Consumption of raw (uncooked) food by mother	Yes	23	34	0.58	0.31–1.08	.09			
	No	77	66						
Consumption of raw (uncooked) food by children	Yes	33	43	0.65	0.37–1.16	.15			
	No	67	57						
Washed produce before eating	Yes	42	57	0.55	0.31–0.96	**.03**	0.57	0.32–1.02	0.06
	No	58	43						
Consumption of street food by mother during past week	Yes	30	17	2.09	1.07–4.11	**.03**	2.06	1.03–4.12	**0.04**
	No	70	83						
Consumption of street food by children during past week	Yes	31	25	1.35	0.73–2.51	.35			
	No	69	75						
Usage of shared latrine by mother	Yes	25	32	0.71	0.38–1.31	.27			
	No	75	68						
Usage of shared latrine by children	Yes	22	31	0.63	0.33–1.18	.15			
	No	78	69						
Availability of latrine in house	Yes	71	66	1.26	0.69–2.29	.45			
	No	29	34						
Used latrine in house	Yes	69	66	1.15	0.63–2.07	.65			
	No	31	34						
Flushed latrine with water after toileting	Yes	69	64	1.25	0.70–2.26	.45			
	No	31	36						
Flooding of toilet in past one month	Yes	6	7	0.85	0.27–2.62	.77			
	No	94	93						

*P* values in bold indicate “statistically significant”.

Abbreviations: CI, confidence interval; OR, odds ratio.

**Figure 2.**
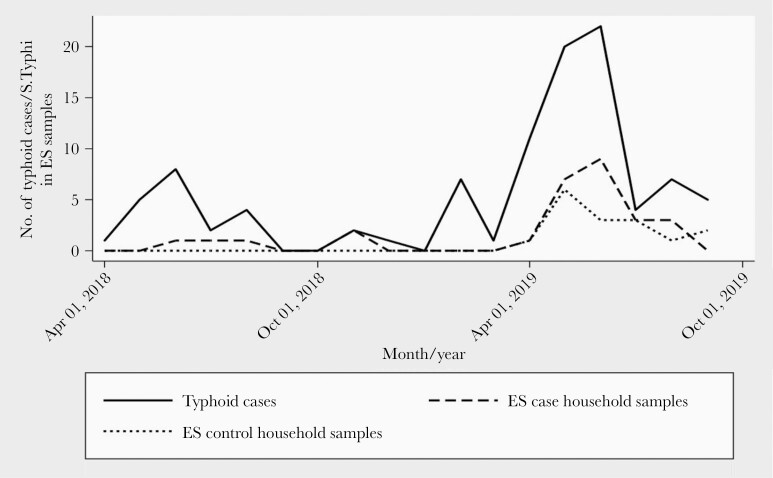
Seasonality of typhoid disease and environmental detection of *Salmonella* Typhi in the households of cases and controls during April, 2018 to October, 2019. ES, environmental sample.

A total of 1097 environmental samples, with 567 and 530 samples from households of cases and controls, respectively (Table 2), were collected. Overall, 4.0% (44 of 1097) of environmental samples were positive for *S* Typhi from households of cases and controls. A total of 4.9% (28 of 567) environmental samples were positive for *S* Typhi in households of cases, compared to 3.0% (16 of 530) in control households (OR = 1.67; 95% CI, 0.89–3.12; *P = *.11). The positivity rate for *S* Typhi was highest for the Moore swab in the sewage samples (16.7% [16 of 96] in cases vs 13% [13 of 100] in controls; OR = 1.34; 95% CI, 0.61–2.96; *P = *.47), followed by BMFS (10.1% [8 of 79] in cases vs 2.6% [1 of 38] in controls; OR = 4.17; 95% CI, 0.50–34.61; *P = *.19) (Table 2). No *S* Typhi was detected in the hand rinse samples of mothers and children (Table 2). The highest proportion of blood culture-confirmed cases of typhoid were detected during April–June 2019 (Figure 2). The peak in *S* Typhi positivity in environmental samples was observed during May–August 2019 (Figure 2).

**Table 2.** Proportion of Household Environmental Samples Positive for *Salmonella* Typhi in Cases and Controls Using Real-Time PCR Assays (Singleplex and Multiplex)

Type of Sample	Case/Control (No. Samples Collected)	Singleplex qPCR Only	Multiplex qPCR Only	Positive by Both Assays	Total N (%)	OR	95% CI
Moore swab (Sewage)	Case (96)	4	3	9	16 (16.7)	1.34	0.61–2.96
	Control (100)	3	2	8	13 (13)		
BMFS (Sewage)	Case (79)	4	0	4	8 (10.1)	4.17	0.50–34.61
	Control (38)	0	1	0	1 (2.6)		
Drinking water	Case (100)	1	1	1	3 (3)	3.06	0.31–29.95
	Control (100)	1	0	0	1 (1)		
Floor swab	Case (92)	1	0	0	1 (1.1)	1	0.06–16.23
	Control (92)	1	0	0	1 (1.1)		
Mother hand rinse	Case (100)	0	0	0	0 (0)		
	Control (100)	0	0	0	0 (0)		
Child hand rinse	Case (100)	0	0	0	0 (0)		
	Control (100)	0	0	0	0 (0)		

Abbreviations: BMFS, bag-mediated filtration system; CI, confidence interval; OR, odds ratio; PCR, polymerase chain reaction; qPCR, real-time PCR.

**Figure 3.**
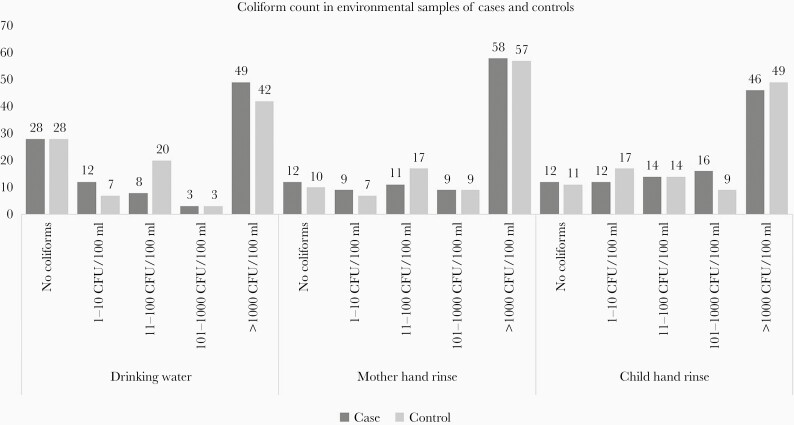
*Escherichia coli* contamination in environmental samples from households of cases and controls. CFU, colony-forming units.

Samples were also tested for *S* Paratyphi A, *S* Typhimurium, *Salmonella* Enteritidis, and a Pan-*Salmonella* target (ttr gene) using a multiplex real-time PCR assay (Table 3), and *S* Typhimurium was found more commonly in the sewage samples in households of controls compared to cases, using Moore swab (14.6% [14 of 96] in cases vs 23% [23 of 100] in controls) and BMFS (2.5% [2 of 79] in cases vs 7.9% [3 of 38] in controls). *Salmonella* Enteritidis was found in approximately 8% of samples in households of cases and controls each. No *S* Paratyphi A was found in the environmental samples (Table 3). A substantial proportion of the drinking water and mother and child hand rinse samples had *Escherichia coli* contamination, with no significant difference between the cases and controls (drinking water, 72% [72 of 100] each in cases and controls; mother hand rinse, 87.9% [87 of 99] in cases and 90% [90 of 100] in controls; child hand rinse, 88% [88 of 100] in cases and 89% [89 of 100] in controls) (Figure 3).

**Table 3.** Detection of Other *Salmonella* Serotypes (Serovars) in Environmental Samples From Households of Cases and Controls Using Multiplex Real-Time PCR Assay

Type of Sample	Case/Control (No. Samples Collected)	Pan- *Salmonella* N (%)	*S* Typhimurium N (%)	*Salmonella* Paratyphi A N (%)	*Salmonella* Enteritidis N (%)
Moore swab (sewage)	Case (96)	56 (58.3%)	14 (14.6%)	0	8 (8.3%)
	Control (100)	73 (73%)	23 (23%)	0	8 (8%)
BMFS (sewage)	Case (79)	20 (25.3%)	2 (2.5%)	0	0
	Control (38)	5 (13.2%)	3 (7.9%)	0	0
Drinking water	Case (100)	23 (23%)	0	0	0
	Control (100)	14 (14%)	1 (1%)	0	0
Floor swab	Case (92)	7 (7.6%)	1 (1.1%)	0	0
	Control (92)	9 (9.8%)	1 (1.1%)	0	1 (1.1%)
Mother hand rinse	Case (100)	1 (1%)	1 (1%)	0	0
	Control (100)	1 (1%)	0	0	0
Child hand rinse	Case (100)	1 (1%)	0	0	0
	Control (100)	2 (2%)	1 (1%)	0	0

Abbreviations: BMFS, bag-mediated filtration system; PCR, polymerase chain reaction.

## Discussion

Our evaluation of risk factors using a case-control approach found treatment of household water protective against typhoid, whereas the mother eating street food was positively associated with typhoid fever in children. Using a molecular approach to detection of *S* Typhi and other *Salmonellae* in the environment, we found a low rate of positivity in sewage samples but no significant difference between case and control households, indicating widespread contamination in this densely populated urban neighborhood.

Our study used real-time PCR assays for the detection of *S* Typhi in the environmental samples. Few studies have discussed the issues related to isolation of *S* Typhi from the environmental samples using culture methods. A study from South Korea found that *S* Typhi enters into a viable, but nonculturable state, in groundwater and pond water and survives longer in that state [15]. Another study from Kathmandu, Nepal reported that culture was not able to detect *S* Typhi in environmental water samples, although *S* Typhi DNA was detected in 77% of the samples using real-time PCR assay [4]. Detection of *S* Typhi in environmental samples using real-time PCR assays have also been recently reported from Bangladesh [16].

There are few case-control studies focusing on the transmission pathways for typhoid fever, especially from Africa and Asia (Table 4). Similar to our findings, street-vended food and beverages have been identified as significant risk factors for typhoid in studies from Uganda, India, and Indonesia [17–20]. Drinking untreated water has been found to be a risk factor for transmission of typhoid in Vietnam, Republic of Fiji, India, and the Democratic Republic of Congo (DRC) [18, 19, 21–24].

**Table 4.** Summary of Case-Control Studies on Environmental Transmission of Typhoid Fever

Sample No.	Place of Study	Year of Study	Age Group	Number of Cases	Number of Controls	Significant Risk Factors for Typhoid Fever	Reference
1	Kikwit, Democratic Republic of Congo	2013	All age groups	320	640	Ever using tap water from the municipal supply, visible urine or faeces in the latrine, knowledge that washing hands can prevent typhoid fever, and stated habit of handwashing habits before cooking or after toileting.	Brainard et al [24]
2	Nairobi, Kenya	2010–2011	All age groups	110	440	Lower elevation of houses associated with increased risk in children <10 years.	Akullian et al [28]
3	Kampala, Uganda	2015	All age groups	33	78	Contaminated water and street-vended beverages.	Kabwama et al [17]
4	Tigray, Ethiopia	2016	All age groups	45	90	Not washing hand after toilet, and unhygienic house and environment.	Mamo et al [26]
5	Blantyre, Malawi	2015–2016	<9 years	125	514	Use of river water for cleaning and cooking, more than 1 water source used in the previous 3 weeks, attendance at school or other daycare.	Gauld et al [25]
6	Mahama, Rwanda	2016	All age groups	260	770	Having a family member who had been infected with *Salmonella* Typhi in the previous 3 months, poor awareness of typhoid fever, inconsistent hand washing practices after use of latrine, eating food prepared at home or at community market.	Nyamusore et al [29]
7	Semarang, Indonesia	1992–1994	≥14 years	75	75	Never or rarely washing hands before eating, eating outdoors at least once a week, eating outdoors at a street food stall or mobile food vendor, consuming ice cubes in beverage in the 2-week period before getting ill, buying ice cubes from a street vendor, less often use of clean water for taking a bath, brushing teeth and for drinking, houses without water supply from the municipal network and with open sewers.	Gasem et al [20]
8	Karachi, Pakistan	1999–2001	<16 years	88	165	Increasing number of persons in the household, nonavailability of soap near hand washing facility, non-use of medicated soap, lack of awareness about contact with a known case of typhoid fever.	Siddiqui et al [5]
9	Mekong delta, southern Vietnam	1996–1997	All age groups	144	144 hospital and 144 community controls	Contact with a patient with typhoid fever.	Luxemburger et al [30]
10	Son La, northern Vietnam	2002	All age groups	90	180	Contact with a typhoid patient, no education, and drinking untreated water.	Tran et al [21]
11	South Dumdum, West Bengal, India	2007	All age groups	65	65	Eating milk products from a particular food handler, drinking piped water.	Bhunia et al [18]
12	Darjeeling, West Bengal, India	2005–2006	All age groups	123	123	Unsafe water, consumption of milk products, unwashed fruits and vegetables.	Sharma et al [19]
13	Kathmandu, Nepal	2011	2–65 years	49	136	Low socioeconomic status, use of stone spout water.	Karkey et al [31]
14	Central Division, Fiji	2014–2017	All age groups	175	349	Interrupted water availability, drinking surface water in the last 2 weeks, eating unwashed produce, having an unimproved or damaged sanitation facility.	Prasad et al [22]
15	Central Division, Fiji	2014–2015	All age groups	80	160	*Escherichia coli* concentrations in toilet drainage soil, drinking water contamination, poor sanitary condition.	Jenkins et al [23]

Our study did not find any significant difference in *E coli* contamination of drinking water samples between households of cases and controls. Similar to our finding, the study from Indonesia found no significant difference in *E coli* contamination of drinking water between the households of cases and controls [20]. However, a study from Central Division, Republic of Fiji, reported a significantly higher concentration of *E coli* in stored drinking water in households of cases compared to controls [23].

The case-control studies from Asia and Africa have reported other risk factors for transmission of typhoid, which includes use of river water for cooking and cleaning, attendance at school or other day care, unhygienic house environments, not washing hands after toileting, contact with a typhoid case, no education, etc (Table 4) [21, 25, 26]. The case-control study from DRC reported an interesting finding of increased risk of typhoid in people with knowledge about handwashing and practicing handwashing before cooking or after toileting [24].

The advantage of using BMFS is to filter a large volume of water in the field without using a power source, thus removing the need for transport of waste water samples to the laboratory. The use of large volume of water also increases the chance of detection of the pathogens in the environment. The use of BMFS in environmental surveillance has been reported to increase the sensitivity of poliovirus detection from waste waters in Kenya and Pakistan [11–13]. Recently, BMFS has been used to detect severe acute respiratory syndrome coronavirus 2 (SARS-CoV-2) in waste water [27].

Our study compared the BMFS with Moore swabs for detection of *S* Typhi in household sewage samples. The BMFS detected *S* Typhi in fewer household sewage samples compared with Moore swabs. However, the number of samples tested using BMFS in our study was low due to the limited availability of these kits and inadequate quantity of sewage in the drains outside the homes.

Our study had several limitations. The number of household sewage samples collected using BMFS was lower and not uniform for cases and controls. Although eating street food by mothers was found to be significantly associated with typhoid fever in our study, we did not assess the food samples and the hand rinse samples from street vendors in the area for detection of *S* Typhi. Due to the smaller sample size in our study, minor associations might have been masked due to similar sampling environments and matching.

## Conclusions

To conclude, our study provides new insights into the risk factors for typhoid transmission in the Indian setting. Consumption of food from street vendors was found to be a risk factor for typhoid in the densely populated urban areas of Vellore. Implementation of measures that are likely to contribute to the control of typhoid in Vellore includes improvement in sanitation facilities and spreading awareness about the treatment of water in the households before consumption. Further case-control studies involving larger geographical areas and higher sample size should be conducted to evaluate the utility of detecting *S* Typhi in environmental samples, especially in sewage and waste water samples, and whether these data can be used to better understand the transmission dynamics of typhoid fever.

## Notes

***Acknowledgments.*** We thank the families of children who participated in the study, the Christian Medical College study team, and the Bill & Melinda Gates Foundation for funding.

***Supplement sponsorship.*** This supplement is sponsored by the Christian Medical College Vellore Association.

***Financial support.*** This work was funded by the Bill & Melinda Gates Foundation (OPP1159346). S. G. was funded by a Global Infectious Disease Research Training Grant (Fogarty International Center, D43TW007392).

***Potential conflicts of interest.*** All authors: No reported conflicts of interest. All authors have submitted the ICMJE Form for Disclosure of Potential Conflicts of Interest.
